# Primary pleural small cell carcinoma with massive pleural effusion: A rare case report

**DOI:** 10.1016/j.ijscr.2025.111450

**Published:** 2025-05-19

**Authors:** Punam Rabha, Jerryes Pious Wisely, Ashutosh Jain, Vinod Raina, Sunita Ahlawat, Sumanta Das

**Affiliations:** a Department of Medical Oncology, Fortis Memorial Research Institute, Gurugram, India; b Department of Pathology, Agilus Diagnostics Ltd, Fortis Memorial Research Institute, Gurugram, India; c Department of Pathology, North Eastern Indira Gandhi Regional Institute of Health and Medical Sciences, Shillong, India

**Keywords:** Pleural carcinoma, Small cell carcinoma, Pleural effusion, Neuroendocrine tumor, Chemotherapy response

## Abstract

**Introduction and importance:**

Lung cancer is the leading cause of cancer-related mortality worldwide, with small cell lung carcinoma (SCLC) accounting for significant morbidity and mortality. While SCLC typically arises from neuroendocrine cells in the central lung, primary pleural small cell carcinoma is exceedingly rare. Its atypical presentation poses diagnostic challenges, emphasizing the need for early recognition and accurate diagnosis to optimize outcomes.

**Case presentation:**

A 70-year-old male presented with progressive shortness of breath and significant weight loss. Imaging revealed massive pleural effusion. Pleural fluid analysis via pleurocentesis, including cytological evaluation and cell block analysis, revealed features consistent with small cell neuroendocrine carcinoma. Immunohistochemical studies confirmed the diagnosis, with tumor cells positive for pancytokeratin, TTF1, synaptophysin, and INSM1, and negative for Napsin-A, p40, WT1, calretinin, and chromogranin. A Ki-67 proliferation index of >90 % indicated a highly proliferative tumor. Chemotherapy with Carboplatin and Etoposide was initiated, leading to a partial response as evidenced by follow-up PET-CT imaging.

**Clinical discussion:**

Primary pleural small cell carcinoma is an exceptionally rare and aggressive variant of SCLC. Accurate diagnosis relies on cytological and immunohistochemical analyses to differentiate it from other pleural malignancies, particularly mesothelioma. The high Ki-67 index indicates its aggressive nature. Management follows conventional SCLC protocols, with systemic chemotherapy as the primary treatment modality. The patient's partial response highlights the potential for disease control with prompt intervention.

**Conclusion:**

This case highlights the rarity and aggressive nature of primary pleural small cell carcinoma. Advanced diagnostic techniques and timely treatment are critical.

## Highlights

• Unlike typical SCLC, which originates in the central lung, primary small cell carcinoma of the pleura arises in the pleura
• Effusion cytology with cellblock is an effective tool to diagnose this aggressive tumor type accurately.
• Early diagnosis is the key to management as it shows a very aggressive clinical course.

## Abbreviations

TTF1: Thyroid Transcription Factor 1
INSM1: Insulinoma-asociated protein 1
WT1: Wilms' Tumor gene 1
PET-CT: Positron Emission Tomography Computed Tomography
INI1: Integrase Interactor 1
BRG1: Brahma Related Gene 1

## 1 Introduction

Lung cancer was the most commonly diagnosed cancer in 2022, contributing to nearly 2.5 million new cases, equivalent to one in eight cancers worldwide (12.4 % of all global cases) [1]. In 2022, the global incidence of small cell lung carcinoma (SCLC) was estimated at approximately 2,480,675 new cases, with an age-standardized incidence rate (ASIR) of 23.6 per 100,000 individuals [1]. The estimated mortality reached 1,817,469 deaths, corresponding to an age-standardized mortality rate (ASMR) of 16.8 per 100,000 people. Small cell carcinoma is one of the deadliest cancers, causing rapid mortality [2]. Small cell carcinoma typically arises from neuroendocrine cells within the bronchial epithelium, most often developing in the central region of the lung, near the large airways [3]. Small cell carcinoma arising from the pleura is sporadic, and only a few cases have been documented in the literature [[4], [5], [6], [7], [8], [9]]. Due to its rarity, it occasionally causes diagnostic confusion. Here, we present the case of a 70-year-old patient who presented with massive pleural effusion. Subsequent cytology and cell block analysis revealed features of small cell carcinoma.

## 2 Case report

A 70-year-old male arrived at the emergency department with progressively worsening shortness of breath, persisting for over a week. Radiology images showed massive pleural effusion requiring immediate pleurocentesis and pigtail insertion. The patient had a history of shortness of breath for nine months, associated with a dry cough. He also experienced unintentional weight loss of 11 kg, nausea, and alteration in taste. He denied any chest wall pain or hemoptysis. He is an ex-smoker with a 12 pack-year history and has a past medical history of hypertension, diverticular disease, high cholesterol, and gout.

## 3 Materials and methods

The work has been reported in line with the SCARE criteria [10].

A PET-CT scan was performed. Due to massive pleural effusion, pleurocentesis was carried out with pigtail insertion, and fluid was sent for cytology. Giemsa stain was performed. A cell block preparation was made using the plasma thrombin method. Immunohistochemistry was performed using the following antibodies: Pancytokeratin** (Clone AE1/AE3, 1:50, Thermo Fisher Scientific), TTF1 (Clone 8G7G3/1, 1:200, Thermo Fisher Scientific), Napsin-A (Clone JE39–28, 1:400, Thermo Fisher Scientific), p40 (Clone 11F12.1, 1:500, Sigma Aldrich), Synaptophysin (Clone SY38, 1:100, Thermo Fisher Scientific), Chromogranin (Clone SP12, 1:100, Thermo Fisher Scientific), INSM1 (Clone BSB-123, 1:100, Bio SB), WT1 (Clone 6F—H2, 1:400, Thermo Fisher Scientific), Calretinin (Clone 6B8.2, 1:1000, Sigma Aldrich), INI1 (Clone RM468, 1:100, RevMab Biosciences), BRG1 (Clone GT2712, 1;200, Thermo Fisher Scientific) and Ki67 (Clone 30–9, Ventana, RTU).

## 4 Results

**Fig. 1:**
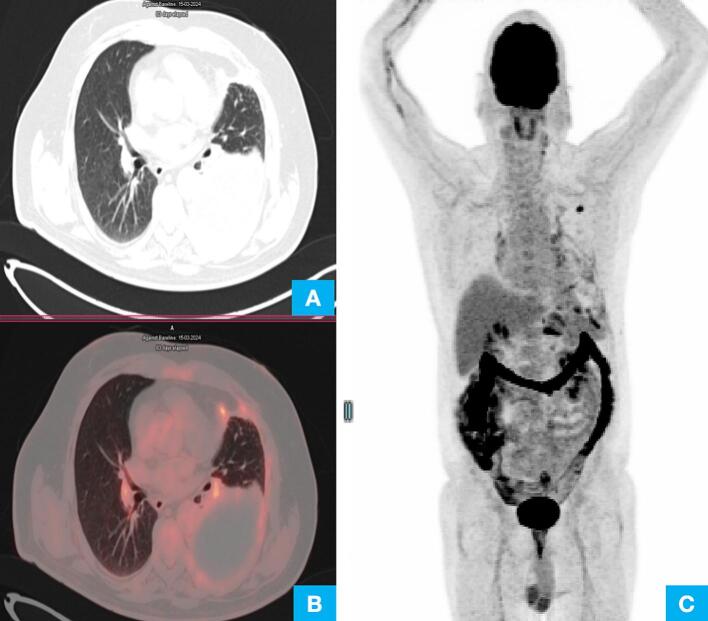
(A) & (B) PET scan shows hypermetabolic left pleural based noduar deposit with gross left sided pleural effusion. (C) Whole body PET scan shows no lung mass and no other mass elsewhere in the body.

The PET-CT scan revealed ill-defined hypermetabolic nodular left pleural thickenings associated with massive left pleural effusion, resulting in the collapse of the underlying lung parenchyma (Fig. 1A, B). Hypermetabolic left internal mammary, anterior diaphragmatic, and coeliac axis lymph nodes were also noted, suggesting involvement. No mass was identified in the lung (Fig. 1C).

**Fig. 2:**
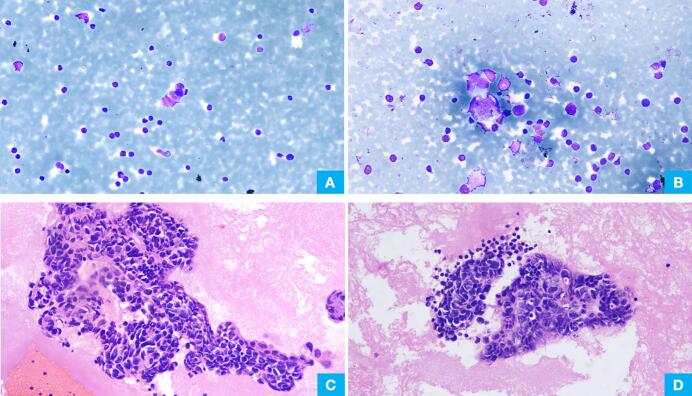
(A) Giemsa stain smear (200×) from pleural effusion material shows lymphocyte rich background with a small clsuter of atypical cells with hyperchromatic nuclei showing molding. Occasional individually scattered atypical cells are also seen. (B) Giemsa stained smear (200×) from other area showing small clusters of atypical cells with hyperchromatic nuclei in a lymphocyte rich background. (C) & (D) Cell block shows nests of tumor cells comprising of tumor cells having scant to moderate cytoplasm, round to oval and spindle shaped nuclei showing nuclear molding.

Pleural fluid cytology was sparsely cellular, showing a predominant population of lymphoid cells with occasional mesothelial cells. Very few atypical cell clusters were seen, showing nuclear molding, with scattered singly lying atypical cells also noted (Fig. 2A, B). Cellblock analysis showed tumor cell nests in a proteinaceous background. Individual tumor cells exhibited scant cytoplasm, round to oval nuclei, and spindling with hyperchromatic nuclei (Fig. 2C,D). Possibilities considered included poorly differentiated adenocarcinoma, basaloid squamous cell carcinoma, and neuroendocrine (small cell) carcinoma.

**Fig. 3:**
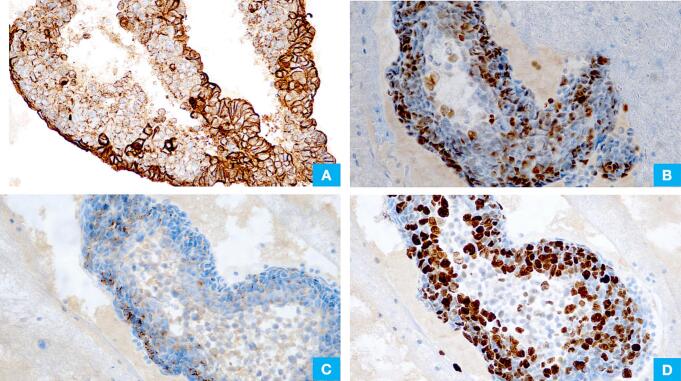
(A) Immunohistochemistry for Pancytokeratin is variably positive. (B) Immunohistochemistry for TTF1 is positive. (C) Immunohistochemistry for synaptophysin is patchy positive. (D) Immunohistochemistry for Ki67 shows high proliferaion index.

Immunohistochemistry performed on the cell block showed tumor cells immunopositive for pan-cytokeratin (Fig. 3A), TTF1 (Fig. 3B), synaptophysin (Fig. 3C), and INSM1 (not shown), and negative for Napsin-A, p40, WT1, calretinin, and chromogranin (not shown). INI1 and BRG1 showed retained nuclear expression (not shown). The Ki-67 proliferation index was >60 % (Fig. 3D). Based on histomorphology and immunohistochemistry, a diagnosis of small cell neuroendocrine carcinoma was rendered.

**Fig. 4:**
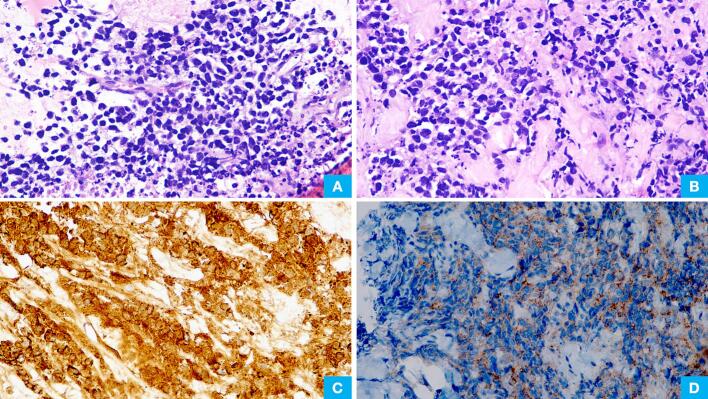
(A) & (B) H&E staining (200×) slides from pleural biopsy showed sheetsof tumor cells comprising of small and medium sized cells having scant cytoplasm, round to oval hyperchromatic nuclei. (C) Immunohistochemistry for Pan-cytokeratin is diffusely positive. (D) Immunohistochemistry for synaptophysin is patchy weak positive.

Pleural biopsy showed sheets of atypical cells comprising small to medium-sized cells with hyperchromatic nuclei and scant cytoplasm (Fig. 4A & 4B). Brisk mitotic activity was noted. Immunohistochemistry for Pan-cytokeratin was diffusely positive (Fig. 4C), and synaptophysin showed patchy weak positivity (Fig. 4D). TTF1 and INSM1 were also immunopositive, and chromogranin was immunonegative (not shown).

The patient was started on chemotherapy (Carboplatin + Etoposide) with palliative intent. After six cycles of Carboplatin and Etoposide, a follow-up PET-CT scan showed an interval reduction in the size and FDG uptake of most pleural base nodules, and a reduction in the volume of left pleural effusion. These findings indicated a partial response to therapy.

## 5 Discussion

Primary pleural small cell carcinoma is exceedingly rare [9]. Small cell lung carcinoma usually presents as a mass in the central airways, such as near the hilum of the lung, or as a mediastinal lesion with accompanying adenopathy [11,12]. In one of the most extensive studies by DC Chieeng et al., only 2.7 % of patients with small cell lung carcinoma developed pleural effusion requiring thoracocentesis [13]. Another SEER (Surveillance, Epidemiology, and End Results) registry reported that 11.6 % of small cell lung carcinoma patients had malignant pleural effusion [14]. Pleural fluid cytology and cell block analysis are valuable tools in diagnosing small cell carcinoma [15].

Clinically, mesothelioma is an important differential diagnosis in cases of pleural thickening or mass presenting with pleural effusion [16]. Morphologically, mesothelioma can show sheets, clusters, and papillae on cytology. On immunohistochemistry, mesothelioma tumor cells are immunopositive for pan-cytokeratin, WT1, and calretinin [17].

The possibility of adenocarcinoma was ruled out by immunohistochemistry. Although small cell carcinoma of the pleura is very rare, it can occasionally present as pleural effusion. As immediate therapeutic intervention is required for this aggressive tumor, awareness of this rare presentation is essential.

Since no cause was found using standard diagnostic methods to indicate that the source of the effusion is from the lungs, it is therefore suggested that the origin may be from the pleura.

## Ethical approval

Ethical approval was waived by the Institute Ethics Committee of Fortis Memorial Research Institute, Gurugram.

## Funding

The study was not supported by any sponsor or funder.

## Author contribution

Conceptualization - Punam Rabha.

Data curation – Punam Rabha, Jerryes Pious Wisely.

Formal Analysis - Punam Rabha, Jerryes Pious Wisely, Sunita Ahlawat, Sumanta Das.

Funding Acquisition - Not applicable.

Investigation – Punam Rabha, Jerryes Pious Wisely, Ashutosh Jain, Vinod Raina, Sunita Ahlawat, Sumanta Das.

Methodology - Punam Rabha, Sumanta Das.

Project Administration - Vinod Raina, Sunita Ahlawat.

Resources - Vinod Raina, Sunita Ahlawat.

Software - Punam Rabha, Jerryes Pious Wisely, Sumanta Das

Supervision - Vinod Raina, Sunita Ahlawat.

Validation - Sunita Ahlawat.

Visualization - Sumanta Das

Roles/Writing - original draft – Punam Rabha, Sumanta Das.

Writing - review & editing – Punam Rabha, Sumanta Das.

## Guarantor

Sunita Ahlawat

## Research Registration Number

Not applicable.

## Consent

Written informed consent was obtained from the patient for publication and any accompanying images. A copy of the written consent is available for review by the Editor-in-Chief of this journal on request.

## Declaration of Generative AI and AI-assisted technologies in the writing process

During the preparation of this work the author(s) used ChatGPT only for two sentences in Conclusion part in order to better representation and make it user friendly. After using this tool/service, the author(s) reviewed and edited the content as needed and take(s) full responsibility for the content of the publication.

## Conflict of interest statement

The authors have no conflict of interest to declare.

## Data Availability

The data that support the findings of this study are available from the corresponding author upon reasonable request.
